# Epidemiology of ebolavirus disease (EVD) and occupational EVD in health care workers in Sub-Saharan Africa: Need for strengthened public health preparedness

**DOI:** 10.1016/j.je.2016.09.010

**Published:** 2017-04-14

**Authors:** Nlandu Roger Ngatu, Ntumba Jean-Marie Kayembe, Elayne Kornblatt Phillips, Joa Okech-Ojony, Masika Patou-Musumari, Mukunda Gaspard-Kibukusa, Ndona Madone-Mandina, Mabasi Godefroid-Mayala, Lubogo Mutaawe, Casimir Manzengo, Dimosi Roger-Wumba, Sayumi Nojima

**Affiliations:** a Graduate School of Health Sciences & Nursing, University de Kochi, Kochi, Japan; b Department of Internal Medicine, Faculty of Medicine, University of Kinshasa, Kinshasa, Democratic Republic of the Congo; c University of Virginia School of Nursing, Charlottesville, VA, USA; d WHO-Ebolavirus Outbreak Response Team, WHO-Liberia, Moronvia, Liberia; e Department of Global Health & Socioepidemiology, Kyoto University School of Public Health, Kyoto, Japan; f Ministry of Social Affairs, Humanitarian Action and National Solidarity, Kinshasa, Democratic Republic of the Congo; g US Agency for International Development (USAID), Kinshasa, Democratic Republic of the Congo; h World Health Organization (WHO), Kinshasa, Democratic Republic of the Congo; i Department of Tropical Medicine, Faculty of Medicine, University of Kinshasa, Democratic Republic of the Congo

**Keywords:** Ebolavirus disease, Epidemiology, Occupational ebolavirus disease (OEVD), Public health preparedness, Work safety

## Abstract

Ebolavirus disease (EVD) is a severe contagious disease in humans, and health care workers (HCW) are at risk of infection when caring for EVD patients. This paper highlights the epidemiologic profile of EVD and its impact on the health care workforce in Africa. A documentary study was conducted which consisted of a review of available literature regarding the epidemiology of EVD, occupational EVD (OEVD), and work safety issues in Sub-Saharan Africa; the literature findings are enriched by field experiences from the authors. EVD outbreaks have already caused 30,500 cases in humans of whom 12,933 died (as of September 9, 2015), and the number of infected HCW has dramatically increased. All eight HCW infected during the 2014 outbreak in Democratic Republic of the Congo died, whereas during the recent West African EVD epidemic more than 890 HCW were infected, with a case fatality rate of 57%. Occupational exposure to blood and other body fluids due to inadequate use of personal protective equipment and needle stick or sharp injuries are among factors that contribute to the occurrence of OEVD. Prevention of OEVD should be one of the top priorities in EVD outbreak preparedness and management, and research should be conducted to elucidate occupational and other factors that expose HCW to EVD. In addition to regularly training HCW to be adequately prepared to care for patients with EVD, it is critical to strengthen the general health care system and improve occupational safety in medical settings of countries at risk.

## Highlights

• Recent EVD outbreaks in Africa (as of September 2015) claimed 12,933 lives.
• Health care workers (HCW) were severely impacted.
• We observed 890 cases of occupational EVD in HCW and a case fatality rate of 57%.
• Use of protective and safety-engineered medical devices should be expanded.
• Periodic training and improving EVD risk communication would enhance preparedness.

## Introduction

Ebolavirus disease (EVD) is a serious infectious disease in humans caused by a virus whose natural reservoirs are thought to be fruit bats of the *Pteropodidae* family.^1^, ^2^ EVD is a deadly disease in humans and can be fatal without proper treatment and care. When an epidemic occurs, health care workers (HCW) are at great risk of infection when caring for EVD patients.^3^ The virus is transmitted to humans through contact with infected living or dead animals, and its propagation in the human population occurs through human-to-human transmission of the virus.^2^ Given that the original EVD epidemic has expanded beyond borders into countries that had been Ebola-free, the disease must be regarded as a global medical emergency.^4^, ^5^, ^6^

The first EVD epidemic occurred in 1976 at a hospital in Yambuku village, province of Equateur, in Zaire (currently the Democratic Republic of the Congo [DRC]), affecting 318 people, of whom 280 died from the disease. The new virus, named ‘Ebola’, after the Ebola River near Yambuku, was simultaneously isolated in blood samples of an infected catholic sister from Yambuku in laboratories based in Kinshasa (DRC), Belgium and at the Centers for Disease Control and Prevention (CDC)/Atlanta in the United States.^7^, ^8^, ^9^

The most recent EVD outbreak started in Guinea in December 2013; it expanded to Liberia and Sierra Leone in May 2014 before reaching other West African countries, including Nigeria and Senegal. This West African EVD epidemic is the deadliest in the history of the disease, affecting at least 28,135 people and killing approximately 11,291 (as of September 9, 2015).^8^ It is the first EVD outbreak to reach other continents beyond Africa, with cases in Europe (Spain, Italy and England) and North America (the United States).^6^ Some of these cases of imported EVD reportedly occurred in people who stayed or worked in western Africa.

In the literature, there are a number of papers and expert opinions that recognized EVD as an occupational disease, but very few have provided potential strategies to address the issue, taking into account the weaknesses of health systems and poor work safety in health care settings in most Sub-Saharan African countries. This paper presents the epidemiologic profile of EVD and the impact of EVD epidemics on the health care workforce and highlights the necessity of reinforcing Ebola disaster preparedness in countries at risk.

### Review of the literature

**Fig. 1:**
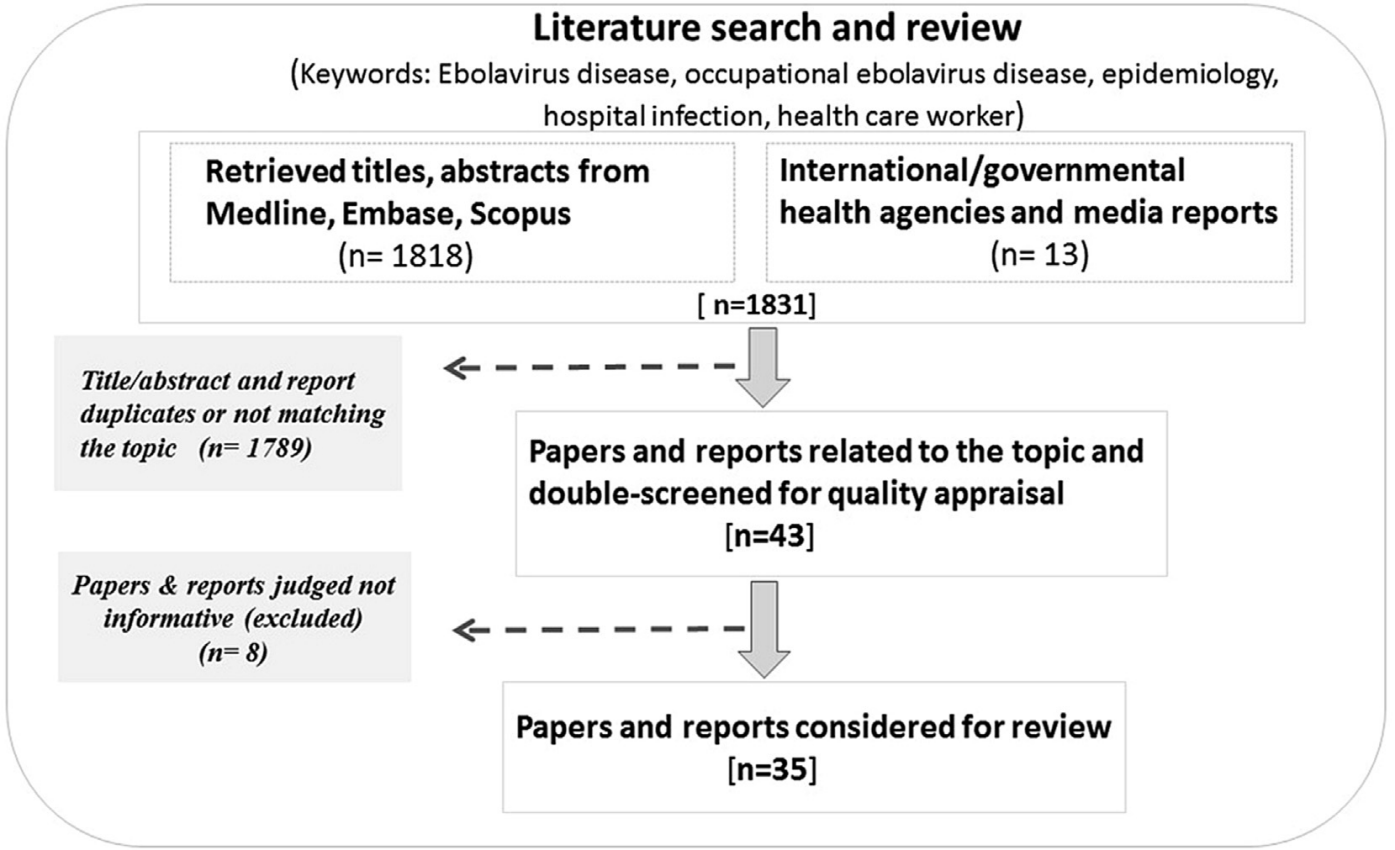
Diagram summarizing the literature review process. Of the 1831 papers, abstracts and reports retrieved from databases, only 34 that were informative and matched the study topic and keywords were considered in this study.

A documentary study was conducted, consisting of a review of the literature regarding the epidemiology of EVD and occupational EVD (OEVD), as well as related work safety issues in Sub-Saharan Africa. This review is enriched by comments and field experiences from authors who worked as Ebola outbreak responders and/or relief care providers in humanitarian settings in affected countries. Medline, EMBASE and Scopus were the scientific databases used to collect articles and abstracts. The following keywords were entered in combination (two or three terms), in the search engines: Ebolavirus disease, epidemiology, occupational Ebolavirus disease, hospital infection, health care worker, work safety, and Africa. In addition, reports on Ebola outbreaks from international and governmental health agencies were also reviewed. This search generated 1831 articles, abstracts and reports; of these, 1797 were excluded after double-screening and selecting only those that were informative and matched the focus of this paper. The final sample comprised 34 articles and reports related to EVD, OEVD, and occupational blood-borne infections (Fig. 1).

### Ebola disease: ecology of ebolavirus, transmission, and historical background

**Fig. 2:**
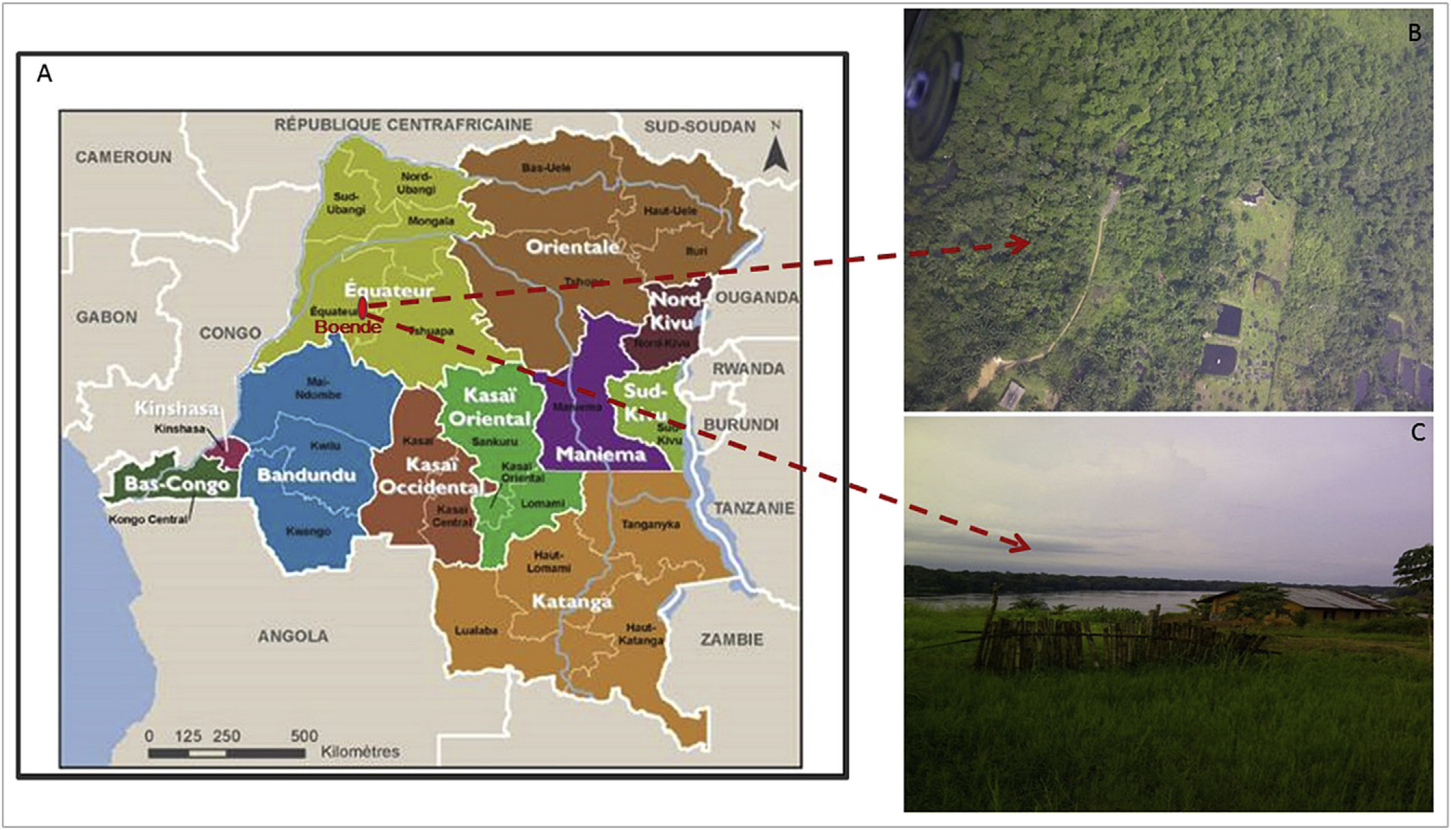
New map of Democratic Republic of Congo (DRC) (A) and location of the epicenter of the recent Ebolavirus disease outbreak (B) and nearby village (C) in Boende.

Source: Authors and Ministry of Social Affairs, Humanitarian Actions and National Solidarity, DRC.

The epicenters of previous and recent African EVD outbreaks all share similar features regarding zones where first cases were found. For example in the Yambuku to Boende (Fig. 2) epidemics in DRC and outbreaks that occurred in Gabon, first cases involved a human contact with wild animals in remote areas located near forests, where bush meat is a favorite food, and poor personal hygiene and sanitation in the community or village are common.

Proximity to the forest, contacts between humans and wild animals and eating habits (i.e., bush meat) are common factors that characterize areas where most EVD related epidemics emerged. In an interview during the West Africa EVD epidemic in 2014, Professor Jean-Jacques Muyembe, a Congolese ebola expert who has investigated 10 Ebola and Marburg disease outbreaks in the central African region since 1976.^10^ stated that in Africa, there are circumstances that typically trigger the transmission of ebolavirus to humans; for example when washing dead bodies during funerals people are likely to get infected, and then they infect relatives. He also mentioned how the outbreak often starts: “hunters go to the forest and find a chimpanzee, gorilla or a dead antelope and cut it into small pieces; they get in contact with blood and get infected. They will contaminate family members and people in the village; when they go to the hospital, given the poor work and sanitation conditions in African rural medical settings, more people get infected and the epidemic will then spread”.^11^

According to a report from the French health organization ‘Medecins Sans Frontieres’ (MSF), the epicenter of the West African EVD outbreak was located around the remote villages of Guéckédu and Manceta in Guinea, which are near forests. The first patients to present symptoms similar to that of EVD were reported by hospital staff and public health services on March 10, 2014.^12^ Similarly, the case of human ebolavirus infection that occurred in Ivory Coast involved contact with a Chimpanzee in a surrounding forest.^13^ These examples suggest the roles played by ecology and the involvement of wild animals in the occurrence of the disease in humans.

It has been suggested that bats could be the reservoir hosts of ebolavirus; however, other researchers think that more evidence is needed to confirm the fact.^14^ The means of transmission of the virus within bat populations remain unclear. An infected bat can transmit ebolavirus to a human, a non-human primate (e.g., chimpanzee or gorilla) or another type of wild animal (e.g., antelope). A human can also be infected through eating meat from an infected animal, and they can then transmit the disease to other humans through contact with blood or other body fluids (BBF) such as urine and saliva.^14^

Since the Yambuku epidemic, several EVD outbreaks have occurred in the central (DRC, Republic of Congo and Gabon), Eastern (Uganda), Northern (Sudan), and Western (Guinea, Sierra Leone, Liberia, and Nigeria) regions of Africa.^15^ The Kikwit epidemic (1995) was the deadliest outbreak to occur in the DRC (formerly Zaire) in a rural city of Bandundu Province, located approximately 530 km from Kinshasa. There were 315 confirmed EVD cases (ten others were clinical cases with negative laboratory test), with HCWs representing 20% of all infected individuals.^6^, ^9^ Given the proximity, the relatively heavy traffic, and the mobility of populations between Kikwit and Kinshasa, the probability of the epidemic reaching the populous capital city was high. However, thanks to the longstanding experience in EVD outbreak response and control by the team of Congolese ebola experts, the expansion of the catastrophe was avoided.

During this Kikwit epidemic, while reinforcing preventive measures in the community to control the epidemic propagation, professor Muyembe's team used blood samples from convalescing patients to treat new cases through transfusion. Of the eight patients treated, seven (87.5%) were saved.^6^ Patients who were recovering from EVD developed antibodies against the virus; these antibodies in the blood improved the condition of transfused patients. This might have been the first anti-ebola immunotherapy trial. The dexterity and savoir faire of the team, combined with the reinforcement and adoption of preventive measures by the members of the affected community, stopped the spread of the Kikwit ebola outbreak.

### Epidemiological profile of ebolavirus disease

Table 1 shows the regional trend of EVD cases and related deaths in Sub-Saharan Africa (Central, Northeast, and West Africa) and western countries. It is estimated that at least 28,177 people have contracted EVD in West Africa, with a case fatality rate of 40%. While the western African EVD outbreak that started in Guinea was expanding in different countries of the region, a new EVD outbreak occurred in Boende, a rural city of the Equateur Province, DRC, in August 2014.^16^ In Central (DRC, Republic of Congo, and Gabon) and Northeastern African regions (Uganda and Sudan), 19 EVD outbreaks have already occurred, with a total of 1522 and 933 cases, respectively. DRC has experienced the greatest frequency of EVD outbreaks, with seven epidemics (1976–2014), followed by Uganda and Gabon.^16^, ^17^

**Table 1:** Number of Ebolavirus disease (EVD) cases and number of EVD-related deaths in humans (1976 to September 9, 2015).

Year	Country	Morbidity	Mortality (*n*; %)
***Central Africa***			
1976	DRC (Zaire)	318	280 (88)
1977	DRC (Zaire)	1	1 (100)
1994	Gabon	52	31 (60)
1995	DRC (Zaire)	315	254 (81)
1996	Gabon	37	21 (57)
1996–1997	Gabon	60	45 (75)
2001–2002	Gabon; Rep. of Congo	122	96 (79)
2002–2003	Republic of Congo	143	128 (90)
2003 (new outbreak)	Republic of Congo	35	29 (83)
2007	DRC	264	187 (71)
2008–2009	DRC	32	14 (45)
2012	DRC	77	36 (47)
2014	DRC	66	49 (74)

**Subtotal**	–	**1522**	**1171 (77)**

***East and North Africa***			
1976	Sudan	284	151 (53)
1979	Sudan	34	22 (65)
2000–2001	Uganda	425	224 (53)
2004	Sudan	17	7 (41)
2007–2008	Uganda	149	37 (25)
2012–2013	Uganda	24	17 (71)

**Subtotal**	–	**933**	**458 (49)**

***West Africa*** (2013–Sept. 2015)	Guinea	3792	2530 (66.7)
	Liberia	10,672	4808 (45.1)
	Sierra Leone	13,683	3953 (28.9)
	Nigeria	20	8 (40)
	Mali	8	6 (75)
	Senegal	1	0 (0)
	Cote d'Ivoire	1	1 (100)

**Subtotal**	–	**28,177**	**11,306 (40)**

***South African region*** (1996)	South Africa	2	1 (50)

**Subtotal**		**2**	**1 (50)**

***Western countries***			
1976	England	1	0 (0)
1996	Russia	1	0 (0)
2013–2014	United states	4	1 (25)
	Spain	1	0 (0)
	Italy	1	0 (0)

**Subtotal**	–	**8**	**1 (12)**

**Total**	–	**30,500**	**12,933 (42.4%)**

DRC, Democratic Republic of the Congo; *n*, number of cases; %, percentage.

Epidemiological data are related to EVD outbreaks from 1976 to September 2015 (DRC Ministry of Social Affairs, Humanitarian Action and National Solidarity; European Centre for Disease Prevention and Control [2015]^16^; World Health Organization^17^).

In the Southern African region, only 2 EVD cases have been reported both in 1996. A few cases of occupational and non-occupational EVD have occurred in or been imported to the western hemisphere, for a total of 8 EVD cases, one of whom died. Taken together, the human impact of all EVD outbreaks is huge, with a morbidity and mortality of 30,500 cases and 12,933 cases (42.4%), respectively (as of September 9, 2015 for the ongoing West Africa epidemic)^17^ (Table 1).

### EVD as occupational disease, work environment in EVD-affected regions and necessity to reinforce public health preparedness

Given the scope of the threat, with increasing morbidity and mortality among HCWs in recent outbreaks, EVD should be considered one of the deadliest epidemics since the beginning of the 21st century, and should receive particular attention from international and governmental health institutions and policy makers. These bodies should consider mobilizing necessary resources to reinforce EVD disaster preparedness and response capacities in exposed populations, with a focus on health care personnel in particular. Reducing the risk of infection among HCWs and volunteers who often are in direct contact with patients in EVD disaster-affected areas should also be one of the top priorities in efforts to counter the spread of the epidemics.

When an EVD outbreak occurs, doctors, nurses, microbiologists and medical technicians are among the first to be deployed to the frontline of the fight against the deadly disease. In addition to educating community members, they are expected to administer health care to infected individuals, which includes manipulating devices that put them in contact with infected BBF. They are ipso facto the most exposed among service providers. This suggests the necessity to implement appropriate and effective preventive measures in order to protect those who care for the communities in affected areas.

**Fig. 3:**
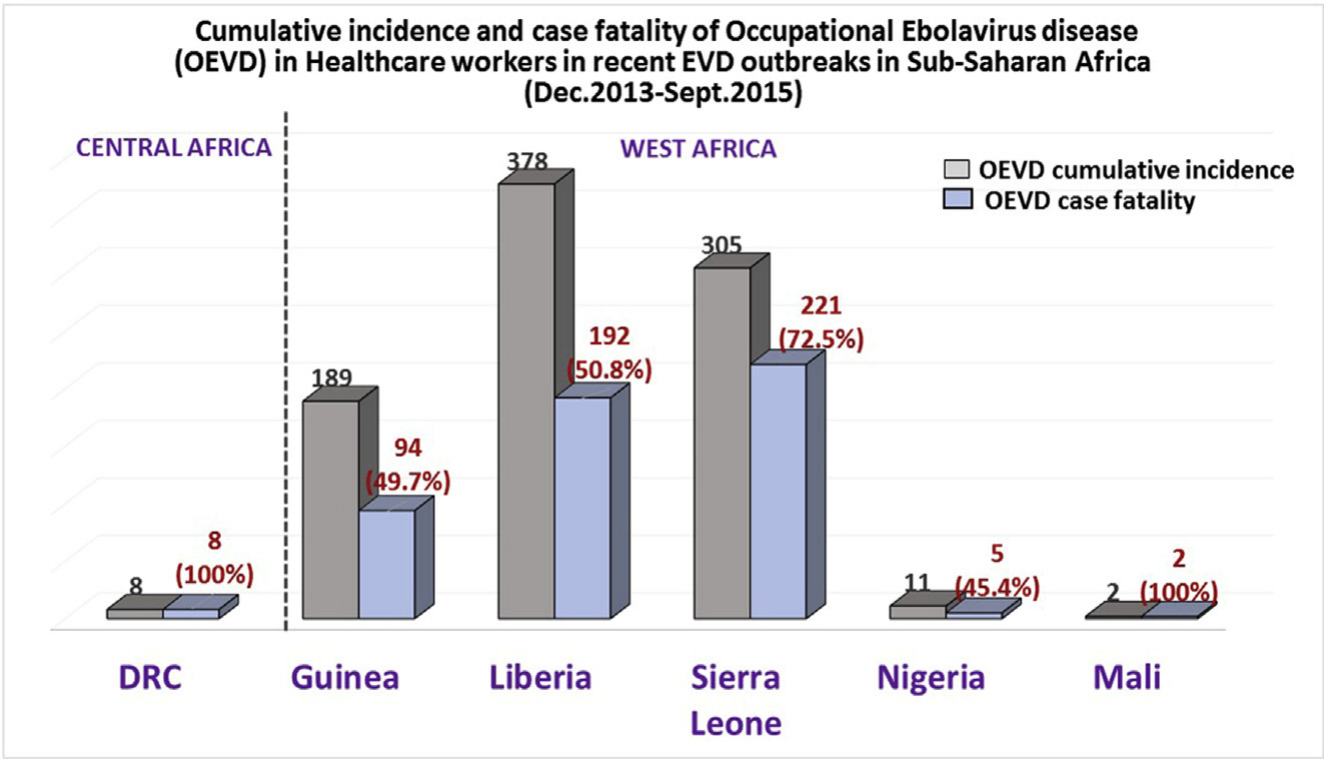
Occupational Ebolavirus disease (OEVD) prevalence and case fatality among health care workers during recent outbreaks in Western and Central African regions. The figure was constructed using data from DRC Ministry of Social Affairs, Humanitarian Actions and National Solidarity, European Centre for Disease Prevention and Control (2015)^16^ and WHO (2015)^17^]. DRC, Democratic Republic of the Congo.

Several reports have shown a considerable number of cases of HCWs who got infected by ebolavirus at their workplace. During the Kikwit outbreak in DRC, for example, 24% of infected individuals were HCW with a case fatality rate of 81%.^6^ In addition, the recent EVD outbreak in Boende, DRC, which occurred a year ago (2014), affected eight medical staff with a case fatality rate of 100%.^18^ Furthermore, the West African ebola disaster, which is the most devastating and deadliest outbreak in the history of EVD to date, has the highest number of infected HCWs. Sierra Leone had the highest incidence (378 cases) and case fatality rate (50.8% [192/378]), followed by Sierra Leone, where there were 305 cases and a 72.5% (221/305) case fatality rate (as of June 30, 2015)^16^ (Fig. 3). It is indispensable to determine and elucidate occupational and behavioral factors that expose health care personnel to EVD, despite safety measures commonly implemented, including the use of personal protective equipment. Identifying and addressing these risky situations will serve to improve work conditions of HCWs and reinforce occupational safety in ebola disaster settings.

Recent EVD outbreaks had a huge psychological impact on both the members of affected communities and those caring for infected individuals. This suggests the necessity for relief care providers to be mentally prepared to respond to such disasters and for them to be taken care of while in the field. One member of the EVD response team in Liberia (one of the authors, JOO), when departing from Uganda to Liberia, described his thoughts about the work environment when caring for EVD patients: “The Ugandan team of HCWs had the biggest number in Liberia, more than 1000, of whom two doctors died in the epidemic. I went to Monrovia (Liberia) alone and it was like going to a place of no return. When we left for Monrovia we had made our wills; I made it three times and tore it up three times and the fourth one went through. As you approach Monrovia, you pray and you pray, and as the planes arrive, you wonder what to expect. People were dying in such a wave; the (health) system broke down, and sometimes there was no knowing who died of what. We were in the CH Rennie general hospital where 27 health care givers died of Ebola. We trained nurses and doctors, set up triage, etc. It was beyond just treating Ebola victims”. When asked what the picture in Moronvia was like, he continued: “it was very bad; what you saw on the news was nothing. There was a time when everywhere you passed, there was death. People were dying in waves … Bodies were rotting on the streets”.^19^ This testimony gives an insight to the psychological context and work environment in which EVD outbreaks responders worked in West Africa.

## Discussion

From the first EVD outbreak to the present, the morbidity and mortality trends suggest that EVD represents a very real disaster for which countermeasures should be adapted to the level of severity of the catastrophe. The number of HCWs infected during recent EVD episodes increased dramatically from past episodes, despite special preventive measures implemented for members of the health care workforce. For a disease that is transmitted primarily through person-to-person contact, it suggests that the work safety conditions among HCW must be optimum in ebola disaster settings.

Occupational safety and health in the Sub-Saharan African countries is still a neglected concept, and percutaneous exposure to BBF, as well as rates of occupational needle stick and sharp injuries among HCWs are high.^20^ Given the danger of exposure to ebolavirus for health care personnel during an outbreak, the World Health Organization, CDC/Atlanta, and other international health agencies and organizations have recently proposed as indispensable the reinforcement of preventive measures for OEVD. The tragic West African ebola epidemic should serve as a wake-up call to better prepare for future epidemics.^21^

Health care personnel should have a feeling that their work safety conditions are satisfactory and that they can protect themselves in such an environment. They should not only be competent in terms of skills, but also be well trained and adequately prepared to care for patients when an outbreak or epidemic such as EVD occurs.^22^ Of the building blocks of a health system framework, more emphasis is put on ‘service delivery and safety’ which primarily translates to patient safety. However, there is a need for integration of both patient and HCW safety, given the risk of communicable disease transmission the latter group faces.

The Global Health Workforce Alliance has reported a global shortage of 7.2 million health care providers with 83 countries (mostly in Sub-Saharan Africa) facing a HCW crisis.^23^ Given the negative impact of EVD on the health care workforce, interventions addressing OEVD should be implemented as part of strategies designed to tackle the issue of EVD and the health care workforce shortage as well. Thus, making safety of HCW explicit in the health system framework is essential as it directly implies that interventions and endeavors that address HCW safety are an integral part of strengthening the healthcare system. Especially in regions where the number of HCWs is low, the loss of even a single worker from illness or death can be catastrophic for the region.

**Fig. 4:**
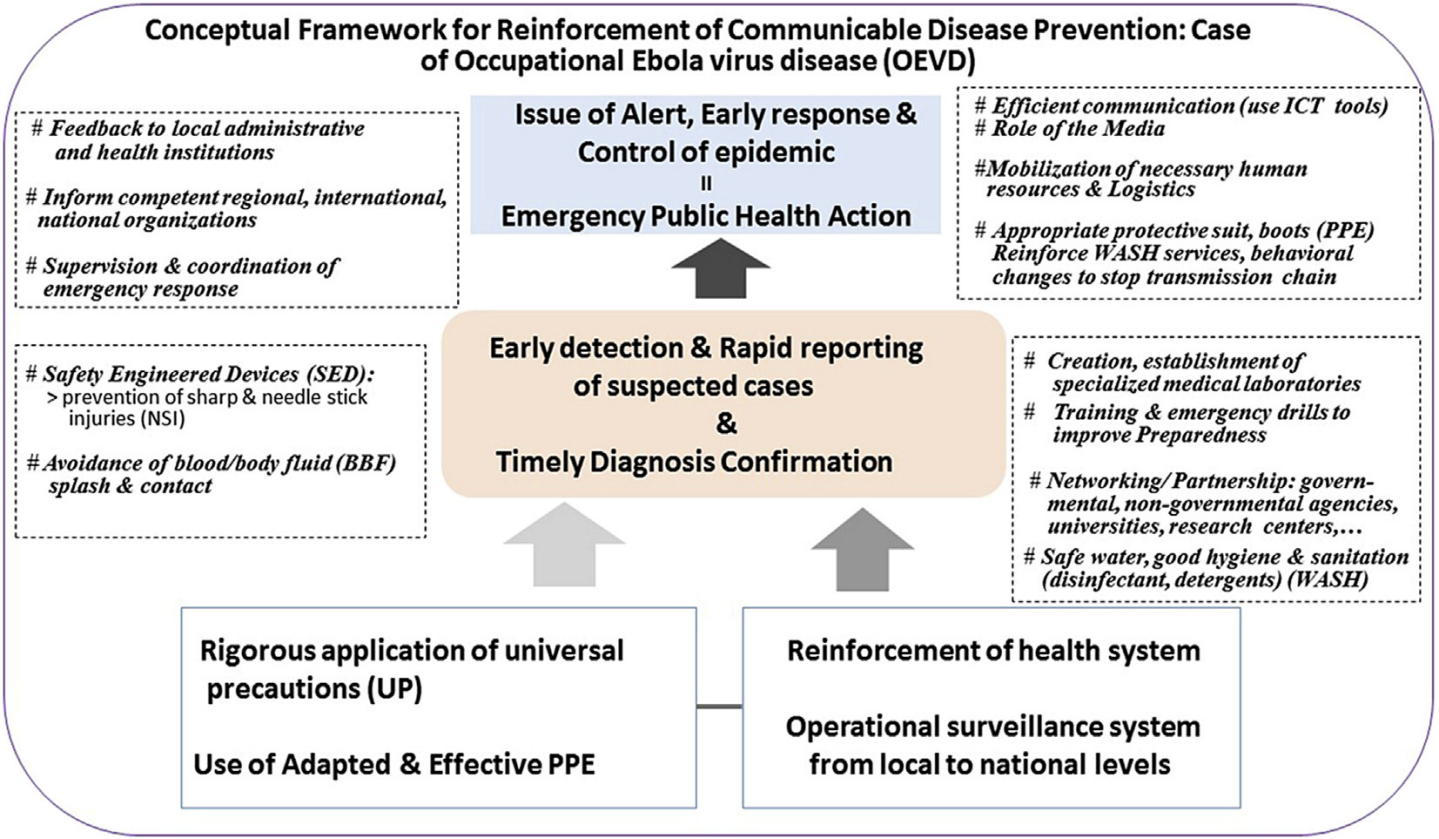
Conceptual framework for reinforcement of work-related communicable disease prevention: case of occupational ebolavirus disease (OEVD). ICT, information and communication technology; NSI, needle stick and sharp injury; PPE, personal protective equipment; SED, safety engineered device; WASH, water, sanitation and hygiene; UP, universal precautions.

In addition, there should be standby counsellors and occupational therapists as part of the team to assist HCW to cope with any eventualities. More importantly, improving preparedness and providing hospitals with necessary laboratory and personal protective equipment, safety devices, and appropriate training to use them, will reduce HCW risk and facilitate early diagnosis of EVD cases and a prompt response (i.e., intervention) (Fig. 4).

### Risk of OEVD and urgency to enhance work safety in health care settings

In the last two decades of the 20th century, the rapid expansion of epidemics of viral infections such HIV/AIDS and hepatitis B and C has created a serious tension in terms of hospital management and safety of personnel exposed in the workplace; it has triggered the adoption of universal precautions for the prevention of nosocomial infections. In most Sub-Saharan African countries, occupational exposure to and infection from BBF is often quite prevalent.^24^ Our previous work has shown that more than 90% of nurses have sustained at least one needle stick or sharp injury in a 12-month period; in addition, all nurses and doctors have been exposed to contact with BBF within the previous year in semi-rural referral hospitals.^20^

A study conducted during the 2000–2001 EVD epidemic in Uganda showed that BBF exposure was the main risk factor that contributed to the spread of the outbreak. Inadequate use of personal protective equipment and needle stick and sharp injuries have exacerbated this risk in the occurrence of OEVD cases both in western and Sub-Saharan African countries.^25^, ^26^, ^27^, ^28^, ^29^ This suggests that occupational safety is a neglected matter, and health policies related to the prevention of nosocomial and occupationally acquired communicable diseases in medical settings should be reinforced and applied at all levels of each country's public health system. Blood-borne pathogens acquired through occupational exposure represent a major hazard and threat for HCWs. In times of outbreaks, they are at risk of exposure, creating a major concern in developing countries, particularly in Sub-Saharan Africa where countries are at high risk of epidemics of communicable diseases and where unsafe practices are common.^30^, ^31^, ^32^, ^33^

Considering the challenges emerging due to the occurrence of new ebola cases in West Africa, following the WHO's declaration of the end of ebola outbreak last December^34^ and the threat of occupationally acquired EVD in recent Central and West African outbreaks, universal precautions and the use of ‘safety engineered devices’ in health care settings should be considered mandatory by international health organizations and local health policy makers in countries at risk of such epidemics. In addition to the promotion of international collaboration in EVD epidemics response, creating a robust local health system that can withstand future outbreaks,^35^ improving sanitation and work safety in hospitals, and establishing an operational surveillance system at local, district and provincial levels should be envisaged (Fig. 4).

In conclusion, it is necessary to strengthen health care systems in countries at risk by adapting triage system in all health facilities, and ensuring strict infection prevention control should be a top priority. This can be achieved through enhancing occupational safety in health care settings by issuing adapted, rigorous, and effective preventive measures, known as universal precautions. Adherence to these safety measures suggests the use of protective equipment (e.g., use of protective garments, an effective protective mask, and goggles), safety engineered devices, proper disposal of contaminated items, efficient handling and management of medical equipment and hospital wastes, and improvement of general sanitation. Furthermore, facilities must implement regular training for health care personnel on safety practices, personal protective equipment and ebola emergency drills at the local level as part of EVD prevention and control to improve ebola disaster preparedness.

## Funding source

The present work was not funded.

## Conflicts of interest

None declared.
